# The diagnostic dilemma of sinusoidal-type primary hepatic angiosarcoma: A case report and literature review

**DOI:** 10.3389/fonc.2025.1704861

**Published:** 2025-11-24

**Authors:** Linfei Dong, Jinghang Xu, Ke Wang, Hui Liu, Li Liang, Huimin Ma, Yan Wang, Na Huo

**Affiliations:** 1 Department of Infectious Diseases, Peking University First Hospital, Beijing, China; 2 Department of Radiology, Peking University First Hospital, Beijing, China; 3 Department of Pathology, Beijing Youan Hospital, Capital Medical University, Beijing, China; 4 Department of Pathology, Peking University First Hospital, Beijing, China

**Keywords:** hepatic angiosarcoma, contrast-enhanced computed tomography, contrast-enhanced magnetic resonance imaging, diagnosis, treatment

## Abstract

**Background:**

Primary hepatic angiosarcoma (PHA) is a rare, highly aggressive, and rapid progressive malignant liver tumor, of which the sinusoidal growth pattern represents one of its uncommon morphological subtypes. Nonspecific clinical presentation, absence of characteristic laboratory findings, and variable imaging features often contribute to diagnostic delays. As a result, patients often miss the window for potential treatment interventions. This study aimed to deepen the understanding of the imaging characteristics of PHA in order to enhance the sensitivity of clinical doctors in identifying rare tumors.

**Case summary:**

A 75-year-old woman presented with poor appetite and progressive jaundice. Initial imaging studies conducted 4 months before admission did not raise strong suspicion for a rare malignant lesion, which contributed to a prolonged viewing time and subsequently led to the decision to perform a liver biopsy. Eventually, immunohistochemical staining of the percutaneous liver biopsy confirmed the diagnosis of sinusoidal-type PHA. Due to her poor baseline condition and critical status, the patient lost the opportunity to receive antitumor treatment and succumbed to the disease within 2 months of diagnosis.

**Conclusion:**

This case underscores the challenges in the early imaging detection of PHA and emphasizes the need for heightened clinical vigilance toward rare liver malignancies. Although histopathology remains the diagnostic gold standard, earlier recognition of suggestive imaging features may prompt a more timely biopsy, enabling prompt treatment and potentially improving outcomes.

## Introduction

Primary hepatic angiosarcoma (PHA) is a rare and aggressive malignant liver tumor that mainly occurs in elderly men in their 60s (1). Although the majority of cases are idiopathic, recognized risk factors include prolonged exposure to vinyl chloride, thorium dioxide, or arsenic (1). PHA poses considerable diagnostic challenges due to its nonspecific clinical presentation and laboratory findings, often leading to reliance on imaging for initial detection. However, the radiographic features of PHA can be complex and variable, which may contribute to delays in the suspicion of malignancy and the subsequent intervention. This case emphasizes the challenge of the diagnosis and treatment of PHA.

In this report, we present a case of sinusoidal-type PHA with a prolonged interval from the initial imaging to the decision to perform a liver biopsy, in part due to the early imaging findings not sufficiently raising clinical concern for a rare malignant tumor. Ultimately, the diagnosis was confirmed histopathologically. However, the patient presented at an advanced stage, with rapid disease progression and poor prognosis. This case underscores the critical need for a heightened imaging recognition and a timely pathological evaluation to improve early diagnosis and treatment of PHA.

## Case presentation

A 75-year-old female patient was admitted to the Department of Infectious Diseases of the Peking University First Hospital in April 2024. She reported a 6-month history of poor appetite, accompanied by progressive jaundice and bilateral lower limb edema over the preceding 2 months. Her medical history included atrial fibrillation on anticoagulation and subclinical hypothyroidism. She denied a family history of associated malignancies or known chronic liver disease. There was no known exposure to vinyl chloride, thorium dioxide, or arsenic.

**Figure 1:**
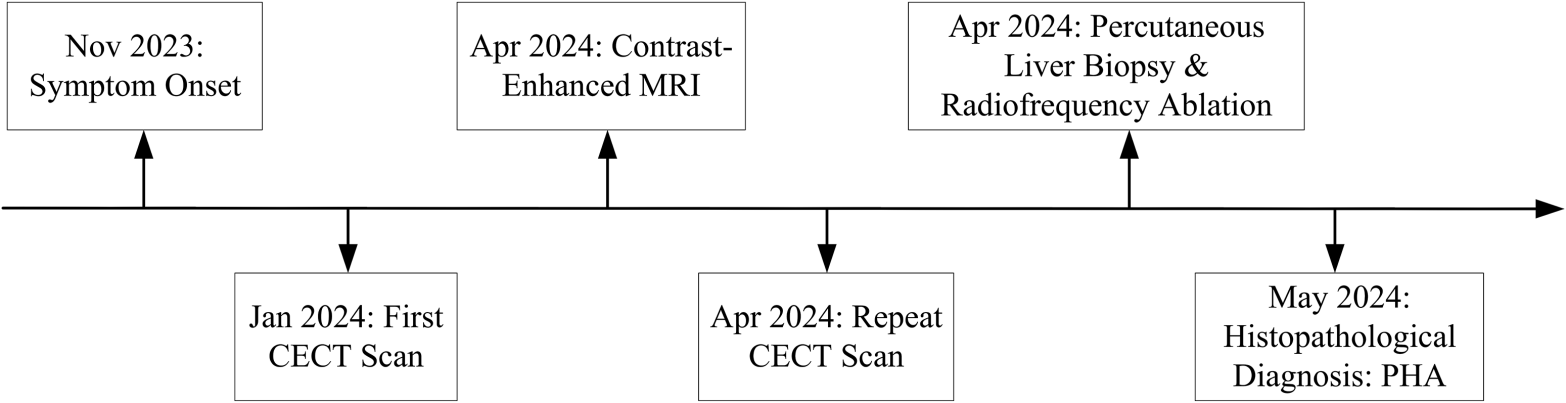
Clinical diagnostic timeline.

Prior to admission to our department, the patient had been evaluated at two different hospitals. A contrast-enhanced computed tomography (CECT) was performed during this period (**Figure 1**). The absence of hallmark radiological signs of malignancy, such as vascular invasion or arteriovenous shunting, led to the exclusion of neoplastic disease. Concurrently, laboratory monitoring demonstrated a progressive increase in total bilirubin (TBIL), a decrease in the albumin (ALB) level, and a decline in prothrombin activity (PTA).

On admission, her laboratory finding was significant for TBIL (136.5 µmol/L), aspartate transaminase (AST, 60 U/L), alanine transaminase (ALT, 100 U/L), alkaline phosphatase (ALP, 170 U/L), and international normalized ratio (INR, 4.49). The patient, on long-term rivaroxaban for atrial fibrillation, was bridged to low-molecular-weight heparin (LMWH) under guidance from the Cardiology Department after admission. Following this transition, PTA showed some recovery, peaking at 60%, but subsequently declined again gradually. Her blood workup for viral hepatitis panel, ceruloplasmin levels, antinuclear antibody, anti-smooth muscle antibody, and anti-neutrophil cytoplasmic antibodies was negative. The levels of carcinoembryonic antigen (CEA) and carbohydrate antigen 125 (CA125) were slightly elevated, whereas the concentrations of alpha-fetoprotein (AFP) and carbohydrate antigen 19-9 (CA19-9) remained within normal limits.

**Figure 2:**
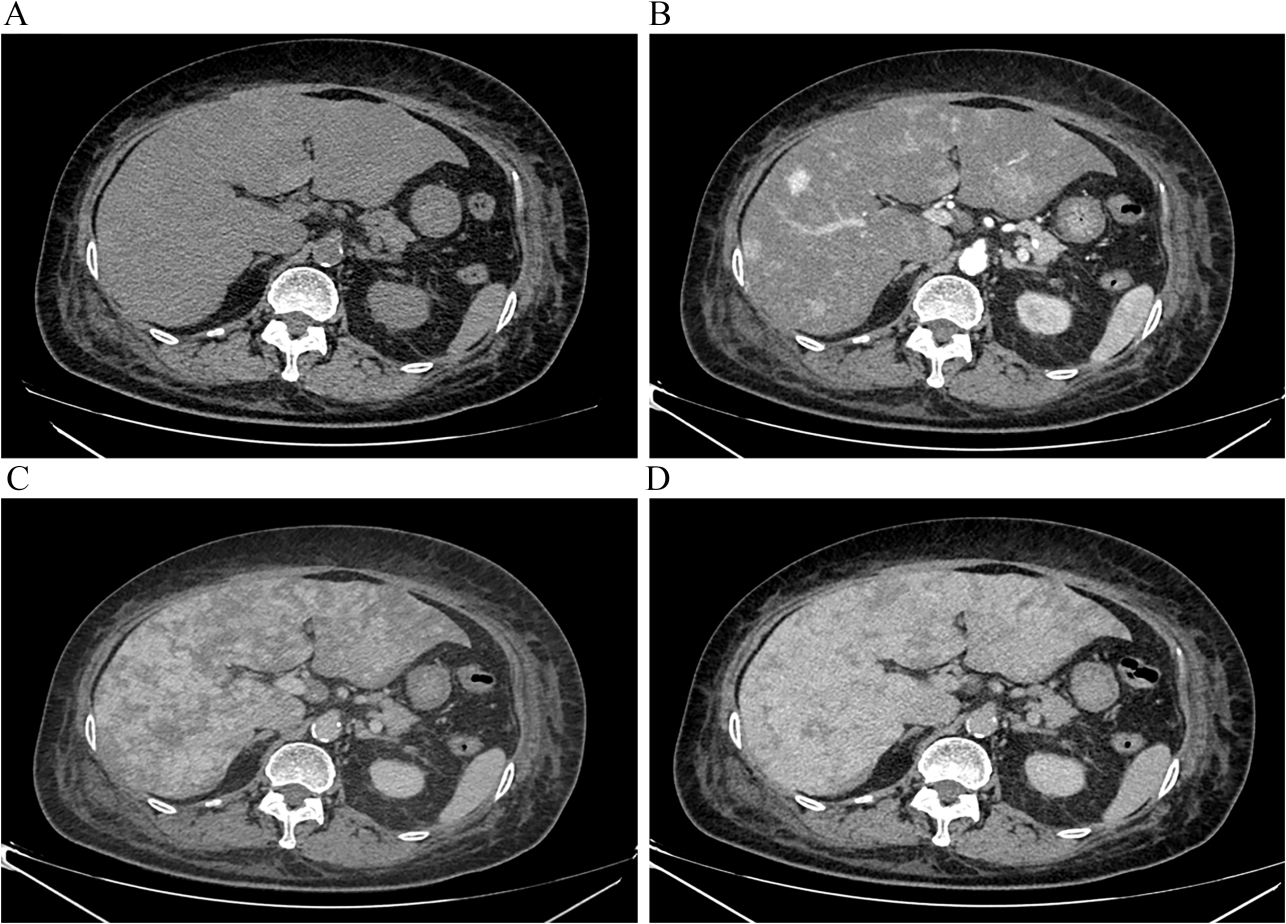
**(A)** Multiple low-density areas shown in the liver before contrast. **(C, D)** Lesions enhanced progressively from the early phase **(B)** to the portal phase **(C)** and almost uniform in the delayed phase **(D)**.

**Figure 3:**
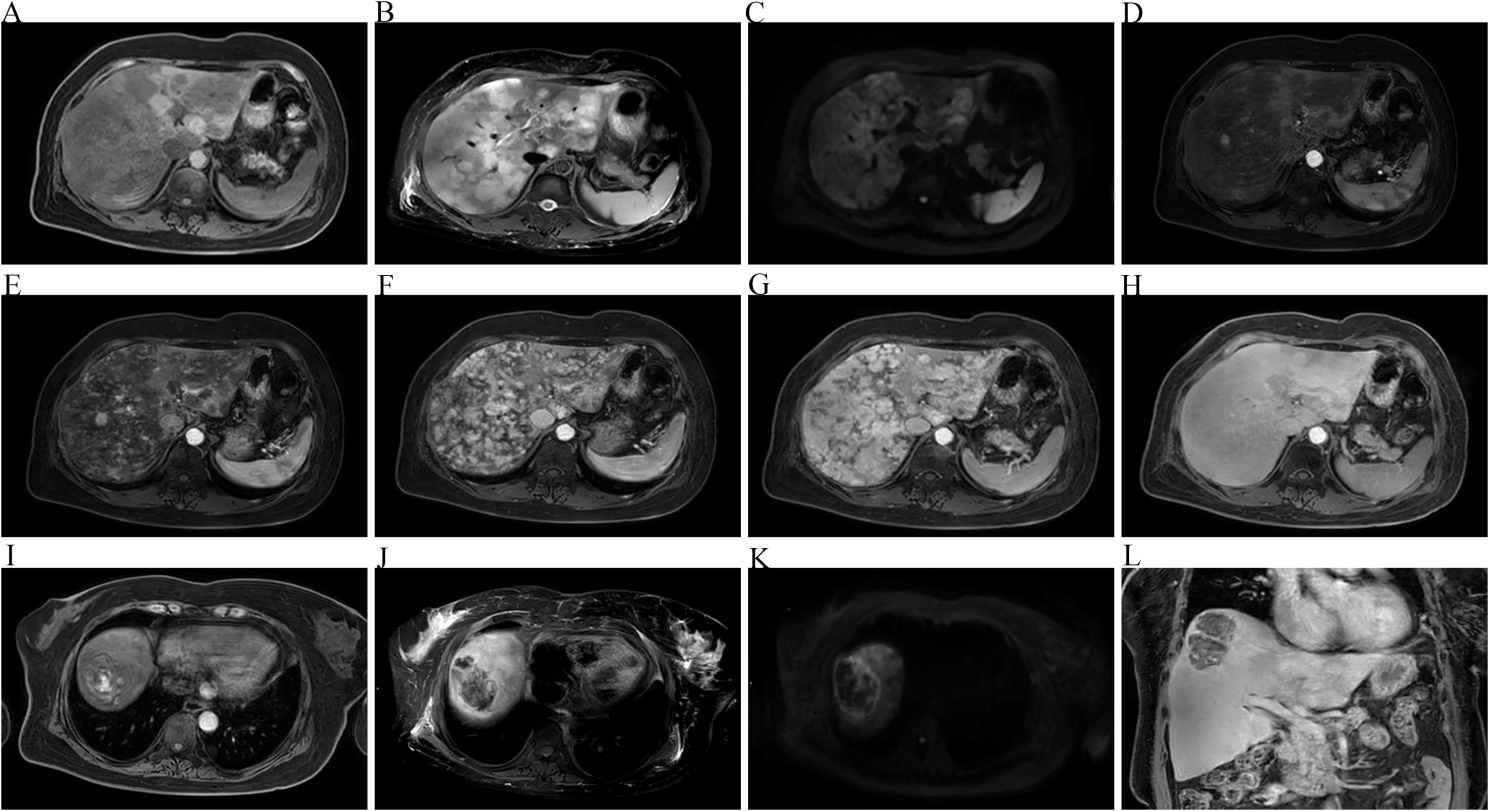
**(A–C)** Multiple lesions showing hypointensity on T1-weighted images (T1WI) **(A)** and hyperintensity on T2-weighted images **(B)** and on diffusion-weighted imaging (DWI) **(C)**. **(D, E)** Lesions heterogeneously enhanced in the arterial phase. **(F, G)** Lesions progressively significantly enhanced in the portal phase **(F)** and the delayed phase **(G)**, with multiple nodular hyperintensity lesions. **(H–L)** No hypoenhancement was evident in the hepatobiliary phase **(H, L)**, except for one hemorrhagic lesion **(I–L)**.

The patient’s deteriorating clinical condition and progressively worsening laboratory findings prompted us to arrange a repeat CECT scan and contrast-enhanced magnetic resonance imaging (MRI) immediately. Imaging studies demonstrated an irregular hepatic surface on non-contrast CT, characterized by multiple nodular and patchy hypodense lesions (**Figure 2A**). These lesions were enhanced from their border in the early phase and to the inside in the portal phase and then almost uniformity enhanced in the delayed phase (**Figures 2B–D**). On non-contrast MRI, these lesions presented as large patchy areas with hypointensity on T1-weighted images (T1WI) (**Figure 3A**) and increased intensity on T2-weighted images (T2WI) (**Figure 3B**), primarily distributed along Glisson’s sheath. The lesions exhibited relatively high signal intensity on diffusion-weighted imaging (DWI) (**Figure 3C**). Gadolinium-ethoxybenzyl-diethylenetriamine penta-acetic acid (Gd-EOB-DTPA)-enhanced imaging revealed partial hyperenhancement on the arterial phase, with delayed and progressive contrast filling (**Figures 3D–G**). No hypoenhancement was observed on the hepatobiliary phase (**Figure 3H**).

**Figure 4:**
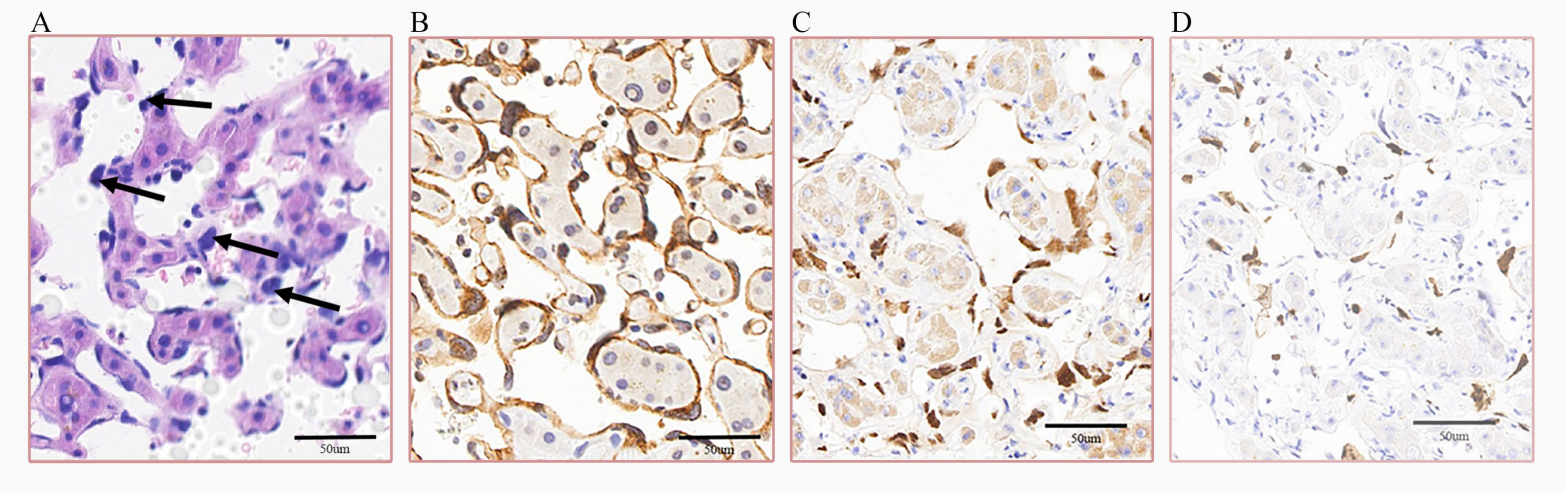
Results of the liver biopsy of the patient. **(A)** Hematoxylin and eosin (HE) staining of the liver tissue (×400) showing that parts of the cells in the sinusoids were enlarged and markedly hyperchromatic. *Scale bar*, 50 μm. **(B–D)** Immunohistochemical staining (×400) of the liver tissue indicating that the cells were positive for CD34 **(B)**, ERG **(C)**, and Ki67 **(D)** (with the Ki67 positive rate approximately 10%). *Scale bars*, 50 μm.

Given the decisive role of pathological examination in determining the nature of the hepatic mass lesions, the patient subsequently underwent percutaneous liver biopsy in the interventional department. Moreover, to further minimize the risk of procedure-related hemorrhage and to achieve local tumor control, radiofrequency ablation was carried out simultaneously following the biopsy. Post-procedural pathological analysis revealed widespread dilation of the hepatic sinusoids and atrophy of the hepatic plates. The sinusoids were lined with large, hyperchromatic, atypical cells (**Figure 4A**). Immunohistochemical staining demonstrated that these cells expressed vascular endothelial markers, specifically CD34 (**Figure 4B**) and ERG (**Figure 4C**), and showed a high proliferation index, as indicated by Ki67 (**Figure 4D**). These findings are consistent with a diagnosis of hepatic angiosarcoma, specifically of the sinusoidal subtype.

Given that the patient’s liver has suffered extensive damage and is accompanied by multiple organ dysfunction, including liver failure and cardiac insufficiency, the multidisciplinary team believes that this patient is not suitable for surgical treatments such as liver lobe resection, chemotherapy, immunotherapy, or liver transplantation. Post-analysis, if the patient had been more vigilant earlier based on the imaging manifestations and had obtained a pathological sample through puncture, she might have had the opportunity to undergo treatment.

## Discussion

PHA, which originates from the sinusoidal endothelial cells of the liver, is an aggressive and rare malignant tumor accounting for approximately 2% of all primary hepatic malignancies (1). It predominantly occurs in elderly men in their 60s. However, there has been a trend of younger onset in recent years. Among the publications over the past 3 years, there were six cases under the age of 14, with the youngest being only 18 years old (2). PHA occurs in association with known chemical carcinogens, e.g., vinyl chloride monomer, radiocontrast colloidal thorium dioxide, chronic arsenic exposure, anabolic steroids, and cyclophosphamide (1). Furthermore, Arif et al. (3) hypothesized that obeticholic acid, a second-line treatment for primary biliary cholangitis, may be an etiologic factor in PHA. However, a recent meta-analysis revealed that fewer than 1% of patients had a history of exposure to these agents (4).

The majority of patients with PHA present with nonspecific symptoms, including abdominal pain, distension, fatigue, poor appetite, weight loss, and jaundice. Some patients, however, may remain asymptomatic until complications occur, such as spontaneous tumor rupture leading to hemoperitoneum and hemorrhagic shock (2, 5), disseminated intravascular coagulation (6), acute respiratory distress syndrome (7), or acute liver failure (8, 9). At diagnosis, distant metastases are usually observed, with the lungs being the most commonly affected, followed by the bones and the spleen (4). Routine laboratory studies often reveal nonspecific abnormalities, such as elevated liver enzymes, anemia, and thrombocytopenia, which lack diagnostic specificity. Furthermore, there are no highly specific tumor markers currently available for PHA, and the levels of tumor markers, such as AFP, CA19-9, CA125, and CEA, are within the normal range in almost all patients.

Imaging studies, which are valued for their noninvasive nature and accessibility, are widely used for the initial diagnosis of PHA. The radiological presentation of PHA can be classified into four main types: multinodular, massive, diffusely infiltrating, and mixed (10). CECT is often regarded as the reference technique. On unenhanced CT, the lesions typically appear as low-density, heterogeneous masses; internal hyperdense areas are mostly suggestive of concomitant hemorrhage (11). Following contrast administration, irregular or annular enhancement is commonly observed in the arterial phase, followed by continuous centripetal filling in the portal and delayed phases (12). MRI, which offers superior soft-tissue resolution, frequently demonstrates heterogeneous signal intensity, including hypointensity on T1WI and hyperintensity on T2WI. Dynamic contrast-enhanced imaging shows increased arterial enhancement; however, the precise enhancement pattern is bizarre and disordered, manifesting as centripetal progression from the tumor periphery, centrifugal enhancement, or a mixed pattern (13). This is followed by rapid and progressive filling during the portal phase and homogeneous enhancement in the delayed phase. Albu et al. (14) concluded that, in patients with PHA, additional T1-weighted scans at 10, 20, and 90 min after gadobenate dimeglumine (Gd-BOPTA) injection can provide helpful information for differential diagnosis, as contrast washout begins at 20 min and completes by 90 min. Notably, Gd-EOB-DTPA-enhanced MRI was utilized for contrast-enhanced imaging and yielded results comparable to those achievable with Gd-BOPTA (15). In general, non-hepatocellular lesions appear hypointense during the hepatobiliary phase. In this case, however, the hepatic angiosarcoma did not exhibit a marked hypointensity in the hepatobiliary phase (20 min), which may be due to the impaired liver function and the abundant sinusoidal components within the lesions.

Although the CT and MRI findings in this case were retrospectively consistent with PHA, they underscore the limitations of relying solely on imaging for the diagnosis of vascular tumors. It is anticipated that continuous advancements in imaging technologies will yield increasingly precise diagnostic information, thereby empowering clinicians to make more informed and proactive clinical decisions in pursuit of definitive histopathological confirmation. In the diagnostic workup of PHA, contrast-enhanced ultrasound (CEUS) and positron emission tomography–computed tomography (PET-CT) serve ancillary roles. In a study of six patients by Wang et al. (16), the US images revealed lesions with heterogeneous internal echogenicity, while CEUS showed heterogeneous hyperenhancement with rapid washout and perfusion defects. PET-CT, while highly sensitive for detecting metastases and guiding biopsy, offers limited diagnostic specificity due to nonspecific 18-FDG uptake overlapping with other hepatic (17, 18).

While imaging studies can aid in diagnosis, pathology and immunohistochemistry are the gold standard methods for the diagnosis of PHA. Histologically, PHA exhibits plump, pleomorphic, polygonal to spindled tumor cells with irregular nuclei, prominent macronucleoli, hyperchromasia, and pathological mitotic figures (19). These malignant cells proliferate infiltratively along preexisting vascular channels such as the sinusoids, replacing normal endothelial cells and forming irregular, anastomosing blood-filled spaces, sometimes resulting in parenchymal loss with associated infarction, atrophy, fibrosis, and vascular occlusion (1). Furthermore, based on a series of 21 cases, Yasir et al. (20) classified PHA into sinusoidal (non-mass-forming) and mass-forming growth patterns, with the latter further subdivided into epithelioid, spindled, and vasoformative subtypes. Immunohistochemically, the tumor cells in PHA demonstrate positivity for vimentin and the vascular lineage markers, including ERG, CD31, CD34, FLI-1, and factor VIII-related antigen (21). Other markers that have been found to be positive in PHA are Ki67 (2, 22), p53 (2), Akt (22), VEGF (22), SMA (23), arginase-1 (23), CK19 (23), AE1/3 (24), BRG (2), CD99 (2), and INI-1 (2, 7). In the presented case, histopathological examination revealed a sinusoidal growth pattern with positive staining for endothelial cell markers, corroborating the diagnosis of hepatic angiosarcoma.

Currently, various therapeutic strategies have been explored in the management of PHA. However, surgical resection and systemic chemotherapy remain the mainstay of treatment. Mangla et al. (25) conducted a retrospective analysis of 364 patients with PHA and demonstrated that both surgical resection (14.6%) and chemotherapy (35.7%) were significantly associated with improved median overall survival [7.7 *vs*. 1.8 months, adjusted hazard ratio (aHR) = 0.23, *p* < 0.0001, and 5.1 *vs*. 1.2 months, aHR = 0.44, **p < 0.0001, respectively]. Another meta-analysis, which included 186 cases, reported a median survival of 15 months for patients undergoing partial hepatectomy compared with 34 months for those with tumors smaller than 10 cm in diameter (26). Although some studies have failed to show a significant survival difference between chemotherapy alone and surgical resection alone, an increasing number of case reports suggest potential benefits from multimodal treatment approaches. Nevertheless, fewer than 20% of patients with PHA undergo surgical intervention, likely due to advanced or non-localized disease presentation, the presence of metastases, or poor baseline conditions at an advanced stage (25, 27). Liver transplantation is contraindicated for PHA due to aggressive disease, high recurrence, and dismal survival, with a reported 12-month survival rate of approximately 24% and 100% mortality by 24 months (28, 29). The efficacy of immune checkpoint inhibitors (ICIs) and targeted therapy remains under investigation. Although the objective response rate of angiosarcoma to ICIs has been reported as suboptimal (30), several case reports suggest that ICIs may represent a potential, albeit non-standard, therapeutic option, and patients treated with ICIs have demonstrated prolonged survival in some observational reports (22, 31).

In conclusion, PHA is a rare and highly aggressive malignancy that presents significant diagnostic challenges due to its nonspecific clinical and imaging characteristics, which can be easily confused with other hepatic tumors. Definitive diagnosis relies on histopathological examination supplemented by immunohistochemical staining. While treatment options are limited, complete surgical resection offers the best chance for cure when feasible. Adjuvant therapies, such as chemotherapy and emerging targeted or immunotherapeutic approaches, may provide additional survival benefits in selected patients. Early and accurate diagnosis is critical to guide timely intervention and potentially improve outcomes in this devastating disease.

## Data Availability

The original contributions presented in the study are included in the article/supplementary material . Further inquiries can be directed to the corresponding authors.
